# The Use of Zebrafish Xenotransplant Assays to Analyze the Role of lncRNAs in Breast Cancer

**DOI:** 10.3389/fonc.2021.687594

**Published:** 2021-05-27

**Authors:** Cecilia Zampedri, Williams Arony Martínez-Flores, Jorge Melendez-Zajgla

**Affiliations:** ^1^ Functional Genomics Laboratories, Instituto Nacional de Medicina Genomica, Mexico City, Mexico; ^2^ Departamento de Ecología de Agentes Patógenos, Hospital General “Dr. Manuel Gea González, Mexico City, Mexico

**Keywords:** lncRNAs, breast cancer, xenotransplant, zebrafish, non-coding RNAs

## Abstract

Breast cancer represents a great challenge since it is the first cause of death by cancer in women worldwide. LncRNAs are a newly described class of non-coding RNAs that participate in cancer progression. Their use as cancer markers and possible therapeutic targets has recently gained strength. Animal xenotransplants allows for *in vivo* monitoring of disease development, molecular elucidation of pathogenesis and the design of new therapeutic strategies. Nevertheless, the cost and complexities of mice husbandry makes medium to high throughput assays difficult. Zebrafishes (*Danio rerio*) represent a novel model for these assays, given the ease with which xenotransplantation trials can be performed and the economic and experimental advantages it offers. In this review we propose the use of xenotransplants in zebrafish to study the role of breast cancer lncRNAs using low to medium high throughput assays.

## Introduction

### Breast Cancer

Breast cancer is the most frequent malignancy in women worldwide and the leading cause of malignancy-related death (1). Whereas early breast cancer is considered curable in 70 to 80% of patients, metastatic disease is considered incurable with our current therapeutic options. The tumor characteristics that lead a breast cancer to become metastatic are not fully understood; however, great efforts are currently being made to elucidate the early mechanisms involved in metastasis, and find early molecular markers and new therapeutic targets related to progression. This malignancy is a heterogeneous group of diseases that deserves our attention and focus on finding new markers that can better discriminate among different subtypes and/or to individualize molecular characteristics of these tumors, allowing for a more reliable prognosis and precise treatments (2). Gene expression profiling of breast cancer has improved the understanding of breast cancer’s heterogeneity on the genomic level, challenged the current classification of breast cancer, served as an important prognostic indicator; and most important, begun to guide the treatment in women with early breast cancer (2). It is of pivotal importance to find noninvasive biomarkers with high sensitivity and specificity, which can be used for breast cancer detection at an early stage and monitor of response to therapy (3). Recent advances in technologies, such as microarray and high-throughput sequencing, represented a deeper understanding of molecular biology, especially long noncoding RNA (lncRNA).

## The Role of lncRNAs in Cancer Progression

Through a varied repertoire of interactions, lncRNAs are involved in health and disease, through a diverse array of processes such as differentiation and embryonic development (4–6), innate immunity (7, 8) and cancer progression (9, 10). The world of lncRNAs is constantly growing; today, several databases have information on hundreds of thousands of lncRNAs from human and other species (11, 12). Several studies revealed that lncRNAs are key to cancer initiation and progression. Although the biological function and molecular mechanisms of lncRNAs are not known in detail, many lncRNAs are expressed abnormally in cancer.

The expression dynamics of lncRNAs are finely controlled by epigenetic, transcriptional and post-transcriptional regulation. The characteristic tissue-specific expression and low transcription levels of lncRNAS are epigenetically regulated. Transcription of non-coding RNA genes is regulated by central transcription factors that also regulate nearby coding genes; however, some lncRNA may allow their transcription to be unsynchronized with their near mRNAs. Moreover, lncRNAs are also regulated at the post-transcriptional level, including modulation by miRNAs (13).

lncRNAs are involved in a large number of molecular regulatory mechanisms such as chromatin dynamics, gene expression, growth, differentiation, and development. Consequently, they participate in the maintenance of homeostasis, and thus, in several pathologic states. These molecules are transcribed at sizes ranging from 200 nucleotides to several thousand base pairs with little or no translation potential (14). lncRNAs comprise non-coding RNAs (lncRNAs) previously annotated as antisense transcripts, intronic transcripts, processed pseudogenes, lncRNAs (long intergenic non-coding RNAs), and coding-transcript isoforms that do not translate to a functional protein (15–18). These RNAs are transcribed in the cell nucleus and then transported to the cytoplasm to be edited and directed to their final destination to fulfill the function, either in the cytoplasm, nucleus, local organelles (cell-autonomous function) or outside the cell (non-cell-autonomous function) (18).

On vertebrates, lncRNAs are transported from cells into interstitial spaces and body fluid through exosomes, similar to lipids, proteins, DNA, and mRNA (19, 20). Secreted exosomes circulate in different fluids and can be internalized by neighboring cells (in autocrine and paracrine communication) or distant cells (in endocrine communication). They can also be transferred from one organism to another, thus facilitating genetic and epigenetic information exchange between organisms (21).

LncRNAs act at various gene regulation levels, e.g., modulating methylation at the chromatin level (22, 23) or regulating genes through association with activator or repressor complexes at the transcriptional level (23, 24). They also participate in processes of splicing, transport, translation and mRNA decay, as is the case of the versatile lncRNAs MALAT1 (17, 25, 26). In summary, lncRNAs fulfill the functions by their molecular interaction with other biomolecules, including proteins, DNA and several RNA species (mRNA, small RNA and even other lncRNAs).

LncRNAs mediate the interaction between proteins, RNAs, and lipids, not only in physiological situations but also during cancer progression (9). These interactions regulate two key cancer processes: Cancer Stem Cells (CSCs) maintenance and the tumor cells´ interaction with their microenvironment. These characteristics give lncRNAs important features as molecular markers for the diagnosis, prognosis (27), and prediction (28) of cancer. Additionally, circulating lncRNAs have great potential as molecular markers for non-invasive detection since variations in lncRNA expression can be detected in a serum or body fluid sample, avoiding invasive approaches such as tumor tissue biopsies (29). This advantage makes lncRNAs a promising tool on the road to early cancer detection and drug design.

### lncRNAs Involved in Breast Cancer

There is currently an extensive list of lncRNAs associated with breast cancer with either oncogenic or tumor suppressor functions, according to their roles in promoting or inhibiting proliferation, metastasis, invasion, apoptosis, autophagy, inflammation, stemness, and drug resistance (30).

### lncRNAs in Breast Cancer Metastasis

Plenty information has recently been generated for some lncRNAs such as MALAT1, HOTAIR (31) and NEAT1 (32) describing their breast cancer progression and metastasis roles; and although knowledge about lncRNAs and their association with breast cancer metastasis is constantly growing (**Table 1**), much remain to be elucidated. In particular, there is a paucity of information regarding the molecular mechanisms by which lncRNAs exert their function and clinical relevance. One of the first mechanisms required to initiate metastasis is the epithelial-mesenchymal transition (EMT) (48), which paves the way for the migration and invasion of cancer cells from the primary tumor site to distant secondary sites (49). More than a dozen lncRNAs are known to be involved in the EMT of breast cancer cells.

**Table 1:** lncRNAs involved in breast cancer metastasis.

lncRNA	Function	Cancer	Reference
ANCR	Tumor suppressor		(33)
NKILA	Tumor suppressor		(34)
XIST	Tumor suppressor		(35)
Linc00052	Tumor suppressor		(36)
NEAT1	Oncogenic		(37)
Linc-ROR	Oncogenic		(38)
UCA1	Oncogenic		(39)
TINCR	Oncogenic		(24)
BORG	Oncogenic		(22)
LincIN	Oncogenic		(40)
Lnc015192	Oncogenic		(23)
LINC01638	Oncogenic		(41)
ARNILA	Oncogenic		(42)
Lnc-BM	Oncogenic		(43)
MALAT1	Tumor suppressor and oncogenic		(44, 45)
HOTAIR	Tumor suppressor and oncogenic		(31, 46, 47)

### lncRNAs Are Involved in Apoptosis Avoidance During Breast Cancer Progression

It is clear that lncRNAs are involved in a wide range of biological and physiological processes during breast cancer progression. One of these is regulated cell death, in particular apoptosis. lncRNA-Zfas1 is an antisense of the 5′end of the gene encoding the Zfas1 protein, which is localized to the ducts and alveoli of the mammary gland. Deletion of lncRNA-Zfas1 in breast cancer cells resulted in increased cell proliferation with a concomitant reduction of Zfas1 expression (50). Thus, Zfas1 is a novel and potential suppressor of breast cancer.

LncRNA-Smad has recently been identified as adjacent to the mouse Smad7 gene (51). LncRNA-Smad7 expression is induced by TGF-beta in all mammary gland epithelial cells and breast cancer cell lines (51). Deletion of this lncRNA neutralized the antiapoptotic function of TGF-β. This finding suggests a tumorigenic role of this lncRNA. LOC554202 is an additional lncRNAs that have been linked to apoptosis repression through interaction with mir-31 in triple negative breast cancer (52). We envision that there are still much to learn about the role of lncRNAs in the regulation of cell death of breast cancer cells.

### lncRNAs and Autophagy in Breast Cancer

Recent studies have shown that the regulation of autophagy is involved in the progression and recurrence of cancer (53), and in the resistance of breast tumors to chemotherapy drugs (54). So, it is not surprising that lncRNA could play a role in the regulation of autophagy in breast cancer cells (55). For example, recent work has identified an autophagy-related lncRNA prognostic signature (ALPS) model composed of five autophagy-related lncRNAs (MAPT-AS1, LINC01871, AL122010.1, AC090912.1, AC061992.1). These results suggested that the autophagy-related lncRNAs are clinically valuable prognostic biomarkers in breast cancer (56).

### Role of lncRNAs in Inflammation During Breast Cancer

It has recently become widely accepted that the immune system can prevent tumor growth and promote it, through processes grouped in three phases: elimination, equilibrium and escape (57, 58). Elimination is achieved through the identification and destruction of transformed cells by tumor-inhibiting inflammation. This phase is characterized by the infiltration of cells of the innate and adaptive immune system. The escape phase is maintained by tumor-promoting chronic inflammation, mainly involving immunosuppressive cells (58).

NF-κB is a family of proinflammatory inducible transcription factors that are involved in breast cancer progression (59). Several lncRNAs play pivotal regulatory roles in the NF-κB pathway. LncRNA NKILA was first found up-regulated by the inflammatory cytokine TNF-α through the NF-κB pathway in breast cancer. NKILA could directly bind to the NF-κB/IκB complex and inhibit NF-κB signaling from suppressing breast cancer metastasis (60). In another report, NKILA was shown to be up-regulated by TGF-β to block NF-κB signaling, thereby suppressing the TGF-β-induced tumor metastasis in breast cancer (34).

STAT3 is a component of another important pathway that plays a role in inflammation during breast cancer progression, and several lncRNAs (e.g., HOTAIR and Lnc-BM) participate in this process (43, 46). In breast cancer cells, Lnc-BM increased the STAT3-dependent expression of ICAM1 and CCL2, which regulated vascular co-option and recruitment of macrophages in the brain, respectively (43).

### The Role of lncRNAs in the Tumor Microenvironment Crosstalk in Breast Cancer

The tumoral microenvironment (TME) is a complex biochemical and physiological system involved in tumorigenesis and metastasis (61–63). It comprises the cancer cells, extracellular matrix, vasculature, non-cancer cells and the tumor’s acidic and hypoxic microenvironment. The cellular component consists of cancer-associated fibroblasts (CAFs), adipose cells, endothelial cells, cancer stem cells (CSCs), infiltrated immune cells such as T lymphocytes and natural killer cells (NKs), myeloid-derived suppressor cells (MDSCs), and tumor-associated macrophages (TAMs) (64–66). There is evidence that lncRNAs are involved in the communication between tumor and non-tumoral cells required to induce or maintain cancer hallmarks such as proliferation, migration, and metastasis.

One of the best-known examples of the relationship of lncRNAs in the communication between tumor microenvironment and tumor cells is the HOTAIR lncRNA. In breast cancer, TGF-β1 secreted by CAFs up-regulates HOTAIR expression to promote epithelium- mesenchyme transition (EMT) and metastasis (67). HOTAIR inhibits miR-7 in CSCs of MCF-7 and MDA-MB-231 breast cancer cell lines and thus promote the overexpression of SETDB1, STAT3, c-Myc, twist, and miR-9 (46) and repression of E-cadherin (46, 68) to the benefit of the EMT process. HOTAIR also contributes to EMT through regulation of VEGF, MMP-9, β-cantenin and Vimentin (69). Also, in breast cancer HOTAIR up-regulates SNAIL expression, as a master regulator of the EMT pathway (31). HOTAIR also mediates the establishment of the SNAIL/HOTAIR/EZH2 tripartite complex by inhibiting the expression of epithelial genes (such as HNF4a, HNF1a, and E-cadherin) through chromatin remodeling in favor of EMT (70).

Another essential component of the stroma required for EMT and subsequent metastasis is tumor vasculature. There is clear evidence that dysregulation of a group of lncRNAs can trigger changes in endothelial cells that favor angiogenesis and metastasis of breast cancer cells. For example, the lncRNA NR2F1-AS1 promotes breast cancer angiogenesis by activating the IGF-1/IGF-1R/ERK pathway (71). Similarly, overexpression of MEG3 suppresses breast cancer angiogenesis through the AKT pathway (72). M2 macrophage-induced lncRNA PCAT6 facilitates angiogenesis of triple-negative breast tumors through modulation of VEGFR2 (73).

CSCs are a subpopulation of cancer cells that can self-renew and proliferate limitlessly. They may be responsible for cancer initiation, progression, and even treatment resistance (74). Several lncRNAs act by modulating the self-renewal and differentiation of CSCs, such as lncH19 and HOTAIR. LncH19 acts as a lncRNA sponge for miRNA let-7, inhibiting its function and favoring the maintenance of CSCs in breast cancer (75). HOTAIR, on the other hand, also regulates the self-renewal of CSCs in breast cancer, inhibiting miR-34a and thus positively regulating Sox2 (76). Interestingly, lncRNAs can also modulate the development, activation and differentiation of T cells, which have both tumor-promoting and tumor-suppressive functions (77). Regulatory T cells (Treg) are a subset of CD4+ T lymphocytes that contribute to the inhibition of anti-tumor immunity of the TME (78, 79). The lncRNA SNHG1 promotes Treg differentiation, and the knockdown of this long noncoding lncRNA inhibits Treg differentiation through increased expression of miR-448 and indoleamine 2,3-dioxygenase (IDO) inhibition, preventing immune escape in breast cancer (80). LncRNAs also modulate immunosuppression and cancer progression through the regulation of ROS (reactive oxygen species), NO (nitric oxide), and ARG1 (arginase 1) production in MDSCs. MDSCs are generated in the bone marrow and have been shown to promote EMT and play an important role in cancer progression by suppressing the immune response (81, 82).

TAMs are also key players in cancer progression through invasion and metastasis regulation. Two functional types of macrophages have been identified, classically activated macrophages (M1) and alternatively activated macrophages (M2) (65). M1 macrophages participate in the Th1-type inflammatory response and have anti-tumor activity, and M2 macrophages are anti-inflammatory macrophages and have a proto-oncogenic role (65, 83, 84). Recently, several studies have shown that lncRNAs can modulate M2 macrophage polarization and by this induce tumor cell migration and invasion in several types of cancer. The lncRNA associated with breast cancer brain metastases (BCBMs), lnc-BM, was found to be overexpressed in breast cancer cells, and associated with the induction of brain metastasis in murine models (43). In breast cancer, lnc-BM increased JAK2 kinase activity to mediate oncostatin M- and IL-6-triggered STAT3 phosphorylation, promote ICAM1 and CCL2 expression, and mediate macrophage recruitment to the brain and consequently metastasis. lnc-BM and JAK2 promote BCBMs by mediating communication between breast cancer cells and the brain microenvironment. Thus, lnc-BM could be a promising therapeutic target for invasive breast cancer (43).

LncRNAs are a diverse set of molecules that can perform their functions intracellularly, travel free in the extracellular matrix, affect distant cells’ function and even be transported by exosomes during intercellular communication. Tumor-derived exosomal lncRNAs affect the TME by generating changes in the transferred cells, E.g. stromal cells, endothelial cells, macrophages, and mesenchymal stem cells, leading to induction of proliferation, angiogenesis and metastasis (85, 86).

### lncRNAs in Breast Cancer Drug Resistance

Besides its involvement with classic cancer hallmarks, a group of lncRNAs has also been linked to drug treatment resistance. The expression of a diverse array of lncRNAs changes dynamically in response to various drugs contributing to anti-tumor drug resistance through various mechanisms, such as cell cycle arrest, inhibition of apoptosis, DNA damage repair (87–89), EMT (90), transport and internalization of drugs by cancer cells (91, 92), and drug metabolism. lncRNAs involved in breast cancer cell drug-resistance are UCA1 in doxorubicin resistance (93), PANDA in anthracycline resistance (94), ARA in adriamycin resistance (95), CCAT2 in 5-fluorouracil (96), and BCAR4, HOTAIR, and M41 in tamoxifen resistance (97–99). Since lncRNAs aberrant expression is a marker of drug resistance (100), they are potential targets of new therapeutic strategies.

## Zebrafish Xenotransplants to Study the Role of lncRNAs in Breast Cancer

Although studies in cancer cell lines have advanced our knowledge of lncRNAs functions at the molecular level, the use of animal models provides a rich context in which to investigate the phenotypic impact of these molecules in the breast cancer.

The involvement of lncRNAs in the breast cancer tumor phenotype can be modeled *in vivo* by genetic modifications in an animal, altering the expression of a lncRNA and studying the effects on cancer development. However, breast cancer´s multigenic and multifactorial nature requires an integrative approach in which the genetic landscape that drives the development of the disease is present.

Xenotransplantation, which is generated by implanting human tumor cells into an animal host, allows the study of the effects of altering a particular gene in the development of breast cancer through genetic manipulation of human cell lines before transplantation. Mouse xenotransplants were the first to be used, but zebrafish have recently emerged due to the experimental, economic, and visualization advantages they offer. In recent years, several breast cancer cell lines have been successfully xenotransplanted in zebrafish, such as MDA-MB-468 (74), MDA-MB-231 (101–104), MDA-MB-435 (105, 106), MDA-MB-23 (102), HCC1806 (101), MCF-7 (36, 107–109) and BT-474 (106) among others. These experiments allowed the exploration of the participation of various genes in pathways related to proliferation-tumorigenesis, apoptosis (109), macrophage-mediated tumor cell invasion (104), migration-metastasis, angiogenesis, drug resistance, stem cell maintenance (106) and tumor microenvironment crosstalk (102, 103).

Zebrafish xenotransplantation represents a step forward in modeling the complexity of breast cancer tumors, and the involvement of a particular gene in each of the events that accompany cancer, as cells are implanted into a living organism in which many types of dynamic interactions can occur. Xenotransplanted cancer cells do not depend on the artificial addition of nutrients, serum, cytokines, and growth factors. In zebrafish, with all functional organs, tumors can engage in both local and systemic cell-cell interactions, shaping tumor progression. These interactions occur between tumor and host and vice versa, with long-distance communication, allowing recapitulation of cancer features such as cell migration, invasion, metastasis, angiogenesis, and immune evasion that are not possible to observe *in vitro*. When breast cancer tumor cells are implanted, many different zebrafish cells are recruited to the tumor site following tumor instructions (102, 103). The zebrafish xenotransplantation model allows simultaneous single-cell resolution monitoring of tumorigenesis at various steps *in vivo*, including tumor vascularization, localized tumor growth, tumor invasion, and micrometastasis formation. Zebrafish xenotransplantation of breast cancer cells enabled the discovery of a new mechanism of metastatic niche formation, and the roles of macrophages in this process were described. The experimental advantages offered by zebrafish also allowed the discovery that physiological migration of neutrophils controls tumor invasion by conditioning the collagen matrix to facilitate the metastatic niche (102).

Finally, drug sensitivity profiling of breast cancer cells using the zebrafish xenotransplantation model allows the assessment of pharmacokinetics, pharmacodynamics and toxicity in a whole living organism, and in a short time. *In vivo* testing has great advantages over *in vitro* assays. E.g., to produce *in vivo* phenotypes, compounds must be absorbed, reach targets, circumvent elimination, and cannot be too toxic, otherwise the animal will not survive. The complexity of *in vitro* models is given by the experience of the investigator, whereas in *in vivo* models, the complexity is built according to the dynamic instructions and signals of the tumor itself. Zebrafish xenotransplantation also allows *in vivo* evaluation at the single cell level of the cell autonomous and non-cell autonomous effects of a drug on the different hallmarks of cancer (110).

There are several methodological advantages for using zebrafish, such as their rapid and external development, the transparency of their embryos (111), the availability of fluorescent cell reporter lines (112), the ease of genetic manipulation (113), and pharmacological approaches (114). Moreover, its wide range of growth temperatures that allows xenotransplantation experiments to be carried out at temperatures close to human physiological ones. These characteristics make the zebrafish an excellent *in vivo* model to visualize the tumor cell behavior and interactions with the host microenvironment.

In addition to facilitating *in vivo* assays related to the breast tumor itself, zebrafish help study functional aspect related to particular molecules such as lncRNAs in breast cancer hallmarks. Xenotransplantation of breast cancer cell lines in zebrafish makes it possible to study human lncRNAs’ role in the tumor phenotype and microenvironment, giving a comprehensive *in vivo* perspective of the functions of this molecule.

Zebrafish xenotransplant facilitates the study of signaling mechanisms involved at the whole organism level during cancer initiation and progression. Furthermore, there is significant conservation of oncogenes and tumor suppressor genes between zebrafish and humans, so the data obtained from zebrafish are relevant to humans (115). The xenotransplantation platform in zebrafish is also helpful for drug discovery in the context of breast cancer research (116). Zebrafish cell xenotransplantation studies have the advantage of maintaining the effects of the microenvironment in cell communication and cancer progression, even when there are inter species differences.

Zebrafish present ideal characteristics that allow multiple statistically robust experiments to be performed simultaneously; however, the zebrafish xenotransplantation platform is not without limitations. On the one hand, the lack of an adaptive immune response is beneficial for initial transplantation and injection, but could become a limitation for translation of findings, as adaptive immune cells may play vital roles in promoting or inhibiting breast cancer progression and the effects of some treatments (117, 118).

The zebrafish and human genomes are 70% similar based on the conservation of individual genes, including cancer-related coding and non-coding genes. However, zebrafish are not mammalian, so some important pathways in breast cancer tumor development are absent, including BRCA1, p16 (CDKN2A), Leukemia Inhibitory Factor (LIF), oncostation M (OSM) and interleukin 6 (IL6) (119). These absent pathways pose several challenges when studying the functions of these “missing” genes or the pathways in which they play a role. Furthermore, when foreign tissues and cells are introduced into fishes, there is no guarantee that all molecular mechanisms linking the recipient tissue and xenograft are fully conserved, which could affect interactions between host cells and the cancerous xenograft. This issue is especially relevant to the study of breast cancer as there is no orthotopic site in fish. However, it may be possible to “add” the necessary cells or growth signals to mitigate this problem during xenotransplantation, or to “humanize” the fish by creating transgenic animals that express appropriate human growth factors, receptors and/or cytokines, as has been done in mice (120).

**Figure 1:**
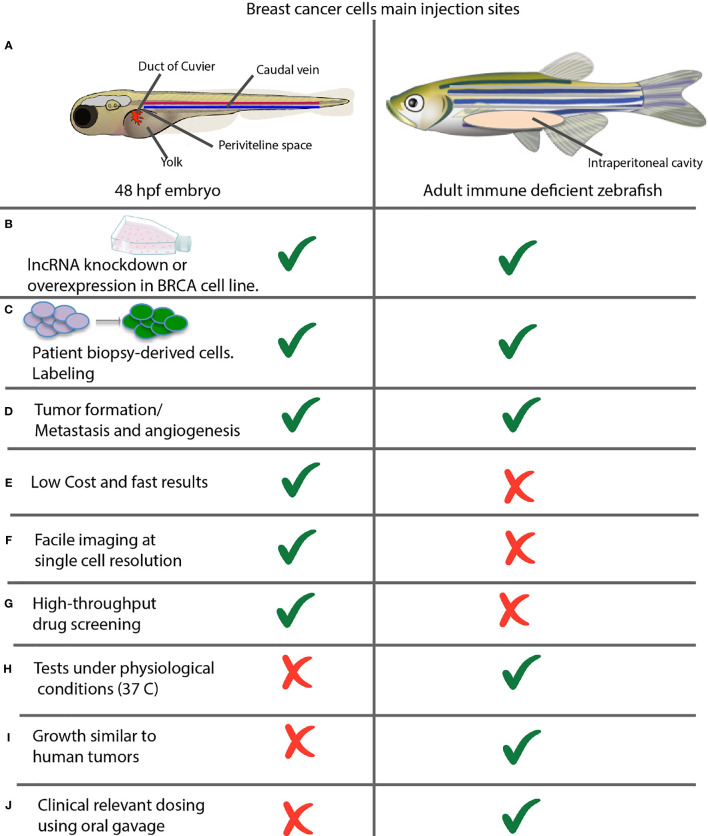
Comparison between xenotransplantation assays in zebrafish embryos and adult animals. **(A)** Common sites of injection. Shown are the most commonly used injection sites for xenotransplantation of a zebrafish in two different stages of development. Left: 48 hpf Stage. The yolk sac is the most common site of injection, but hindbrain ventricle, caudal vein; previteline space and duct of cuvier can be used also. Right: Juvenile Adult. The majority of xenografts occur within the intraperitoneal cavity, and hinbrain ventricle can be used also. **(B)** Xenotransplantation of cancer cell lines and **(C)** patient-derived cells can be performed in both embryos and adults. **(D)** Xenotransplantation allows evaluating the rate of tumor formation, metastasis and angiogenesis. **(E–J)** Advantages and disadvantages of xenotransplantation in embryos versus adult fish assays.

Zebrafish offers two options for cancer modeling by xenotransplantation of breast cancer cell lines or patient-derived tumor cells by microinjection, (**Figure 1**). In 48 hpf (hours post-fertilization) embryos, into the yolk sac, duct of Cuvier (common cardinal vein), caudal vein, or perivitelline space. In adults, into the intraperitoneal cavity, (**Figures 1A–C**). Either option may result in tumor masses, which induce a neo-vascular response around the tumor and consequent migration and metastasis, (**Figure 1D**). Embryo assays are the top choice in most work, given their methodological advantages for fast results, low-cost experiments, compatibility for microscopic imaging, and drug screening potential taking advantage of their small size, (**Figures 1E–G**) (121).

On the other hand, adult animal assays are ideal for studying human physiological temperature-dependent characteristics such as tumor growth rate or determining the dose using oral gavage, (**Figures 1H–J**) (121, 122).

The use of fishes with fluorescent vasculatures such as Tg(*Fli : EGFP*) or *VEGFR2:G-RCFP* allows the visualization of angiogenesis *in vivo* (123–125). In xenotransplantation, the zebrafish provides the necessary signals for the transplanted cells to integrate into the organs, migrate, proliferate and interact with the zebrafish microenvironment (126, 127).

Xenotransplantation has evolved as one of the most valuable strategies to study breast cancer’s discrete aspects, as evidenced by the growing volume of scientific publications on this subject over the last 15 years, (**Supplementary Table 1**). Xenotransplants of breast cancer cell lines in zebrafish allow rapid *in vivo* testing of coding and non-coding genes, pathways involved in tumorigenesis, migration, angiogenesis, or screening for new drugs. Moreover, in recent years, *in vivo* modeling by zebrafish xenotransplantation revealed important information on the role of some lncRNAs in breast cancer hallmarks (36, 107, 128, 129) (**Supplementary Table 1**, blue data). We recently uncovered the role of lncRNA-HAL in promoting the stemness in breast cancer cells; the action of lncRNA LINC00052 in the suppression of migration, as well as the role of LncMat2B in the induction of breast cancer cell invasiveness using *in vivo* xenotransplantation assays in zebrafish (36, 107, 129). Likewise, Peperstrate et al. showed that lncRNA H19 increases breast cancer cells’ invasive capacities in xenografted transgenic zebrafish models (128). In this work, breast cancer cell lines were modified to alter the expression of lncRNAs, then stained or labeled with reporter genes and transplanted into zebrafish embryos. Tumor cells that migrated to distant sites within the fish embryos, and the growth of the transplanted mass, or the development of tumors at secondary sites, were related to the different hallmarks of cancer to infer the involvement of the lncRNA ones in these events.

In conclusion, zebrafish xenotransplants allow the *in vivo* functional study of the involvement of lncRNAs in breast cancer in short timescales.

## Zebrafish Xenotrasplant for the Study of lncRNAs in Breast Cancer Tumor Microenvironment

One of the most attractive advantages of using an *in vivo* model for the study of breast cancer as a complement of an *in vitro* model is the possibility of representing the complex context of the tumor microenvironment.

As discussed above, lncRNAs are involved in a various molecular pathways related to communication from the tumor microenvironment to the tumor cells themselves to promote cancer establishment and progression. The zebrafish xenotransplantation platform for breast cancer will facilitate the discovery of functional information of lncRNAs in the complex process of communication with the tumor microenvironment. Zebrafish xenotransplantation allows visualization of *in vivo* events in a real time and cellular level, such as cell-cell interaction. Together with assays to alter the expression of lncRNAs in xenotransplanted cells, the zebrafish xenotransplantation model could provide valuable information on the participation of lncRNAs in this complex process. As previously stated, zebrafish xenotransplantation models are efficient for providing information about several breast cancer hallmarks, e.g. tumor progression, angiogenesis, spread, metastasis or drugs response, revealing the existence of interactions between cancer cells and cellular and non-cellular components of the host inter-species microenvironment.

Xenotransplantation assays in zebrafish have shown that it is possible to investigate the mechanisms and biological implications of tumor-host cell crosstalk. A clear example is the molecular interaction between breast cancer cells and zebrafish host cells. They allow the recapitulation of cancer hallmarks such as angiogenesis in CXCR4 chemokine signaling across zebrafish and humans in xenotransplantation experiments. Tullota et al. showed that human cancer cells expressing CXCR4 responded to the zebrafish Cxcl12 ligand, and zebrafish cells expressing Cxcr4 migrated to the human CXCL12 ligands (130). On the other hand, substantial evidence supporting the molecular interrelationship between human and zebrafish, and involving a lncRNA, is the resistance to tumor formation of a zebrafish knockout of *Thor* (THOR-/-) (an oncogenic lncRNA conserved between zebrafish and human) after xenotransplantation with NRAS61K melanoma cells (131).

The interaction between cancer cells and zebrafish immune cells was discovered by experiments transplanting cancer cells directly into the blood circulation through the duct of Cuvier or the perivitelline space. Neutrophil and macrophage infiltration surrounding the tumor was observed by using transgenic zebrafish strains with labeling in immune system cells (Tg(mpx:GFP)^i114^ (132) in neutrophils, Tg(mpeg1:eGFP)^gl22^ (133) and Tg(mpeg1: mCherry)^UMSF001^ (134) for macrophages (135); and the interaction of cancer cells with endothelium using vessel-tagged strains such as Tg(fli:eGFP)^y1^ (112), Tg(flk1:eGFP)^s843^ (136) and Tg(flk1:mCherry) (137). The zebrafish immune cells are recruited and localized near the breast cancer cells at the primary tumor growth and secondary micrometastasis sites. Also, it was observed that the non-disseminated tumor cells associated with the endothelium of the duct of Cuvier and remodeled it, forming new vessel-like structures and then forming functional vasculature. Subsequently, by knocking down the expression of myeloid differentiation transcription factors in zebrafish, the suppression of tumor vascularization, invasion and micrometastasis was observed (102), showing the dynamic interaction of zebrafish immune cells with human breast cancer cells.

On the other hand, using vasculature-tagged reporter strains, cancer cells injected into the yolk of zebrafish embryos were shown to interact with the endothelium of the embryos blood vessels, migrate through them and form secondary tumors (138). The induction of angiogenesis mediated by the interaction between zebrafish immune cells and transplanted human breast cancer cells was also confirmed through the positive correlation between the expression levels of vascular endothelial growth factor A (VEGFA) secreted by transplanted breast cancer cell line, the number of immune cells recruited around the tumor, the interaction of macrophages with the vessels, and the induction of new vessel formation around the tumor in zebrafish (103).

In recent work, it was observed that one of the mechanisms by which the interaction between host cells and tumor cells occurs is through the transfer of cytoplasm from zebrafish macrophages to the transplanted tumor cells. Although it is unknown what components are exchanged, it is presumed that it could be RNA molecules (139).

Zebrafish xenotransplantation assays of breast cancer cells, coupled with single cell transcriptional analyses, could facilitate the elucidation of the molecular mechanisms and lncRNAs involved in the communication between the tumor microenvironment and cancer cells.

## lncRNAs and Zebrafish Patient-Derived Xenotransplantation (zPDX) in the Search for Personalized Breast Cancer Treatments

Due to breast cancer’s genomic advances, one of the greatest challenges in translational and personalized medicine is quick, cheap and reproducible *in vivo* disease modeling. Although molecular breast cancer markers and pharmacogenomics analyses help to predict the best treatment option, many patients do not respond as expected. This event is probably due to the heterogeneity of breast tumors, in which there are non-responding cells immersed in a large group of responding cells, which will escape treatment.

LncRNAs have been associated to breast cancer progression by modulating a large number of oncogenic processes. These results point toward the possibility that they could be useful as future targets for therapeutic intervention against breast cancer (129, 140, 141). In addition, Several studies have proposed lncRNA signatures that could potentially be used for predictive and prognostic value in response to breast cancer treatments (142–144).

LncRNAs can be targeted for inhibition through multiple mechanisms, such as antisense oligonucleotides (ASOs), short hairpin RNAs (shRNAs), short interfering RNAs (siRNAs), aptamers, CRISPR-Cas approaches and small molecule inhibitors (145, 146). There is evidence that ASOs could be a potential targeted therapy for cancer-associated lncRNAs (147). It was recently reported that ASOSs directed against the breast cancer-associated lncRNA MALAT1 effectively suppressed cancer spread to the lung in a murine model of breast cancer xenotransplantation (148).

**Figure 2:**
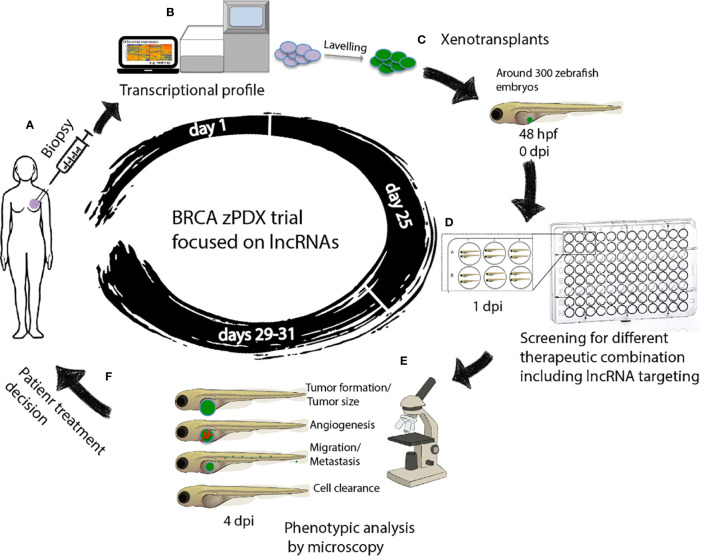
Workflow of zAVATARS in the context of personalized medicine. **(A–C)** Experimental setup for generating zebrafish xenotransplant models. Cells derived from itumor biopsy are analyzed by RNAseq, subsequently labeled and microinjected in the 2dpf larvae. **(D)** One day after injection, larvae are screened for successful injection and distributed in groups for testing chemo-, and/or biological therapies. **(E)** Three days after treatment, larvae are processed for in vivo microscopy for analysis of proliferation, cell death, angiogenesis, and metastatic potential. **(F)** Treatment decision of breast cancer patients based on response of zebrafish cancer hallmarks in drug screening. hpf: hours post fertilization. dpi: days post injection. BRCA zPDX trials: Trial of patient-derived xenotransplants in zebrafish for the study of breast cancer.

The use of avatars or patient derived xenotransplantation (PDX) breast cancer models may help evaluate *in vivo* the global response of tumor cells, detect those that escape the drug, and find and decide the most appropriate treatment for that patient. Zebrafishes offer suitable characteristics for breast cancer modeling using PDX, such as the embryo transparency, the possibility of real time visualization, and the short time to obtain results (149). Patient derived xenotransplantation in zebrafish (zPDX) consists of obtaining a very small fraction of the patient’s tumor by biopsy and transplanting it into zebrafish directly or after obtaining a primary culture of cells, (**Figures 2A, C**).

The combination of zPDX with the lncRNAs based transcriptomic analysis-guided drug screening assays would enable the finding of efficient and personalized anti-breast cancer treatments, (**Figures 2B–D**). The zebrafish xenograft model allows rapid sensitivity profiling to new anticancer drugs but is also ideal for determining the effects of different therapeutic combinations on tumorigenesis, metastasis, and angiogenesis, in a timeframe compatible with the clinical decision-making process (110), (**Figures 2D–F**). More important, these assays maximize the use of the small amount of breast cancer tissue available after a biopsy, which can be a limiting factor in precision medicine.

## Zebrafish Conserved lncRNAs in the Future Directions for Breast Cancer Modeling by Xenotransplantation

LncRNAs are key players in the communication between tumor cells and the surrounding microenvironment, actively participating in cancer progression. These non-coding RNAs can travel free or *via* exosomes to neighbor cells in the tumor microenvironment and carry out their function in a cell-non-autonomous manner (19). Breast cancer xenotransplantation assays in zebrafish showed that there is interspecies molecular communication that allows the development and progression of cancer in the animal model. Despite the low conservation of interspecies sequences of lncRNAs, the knowledge of those that are conserved between human and zebrafish will allow the study of their cell-non-autonomous function, and to test their potential as therapeutic targets.

LncRNAs can be evolutionarily conserved through sequence, structure, function, and expression of the locus of synthesis. In general, lncRNAs do not have high sequence conservation across the full-length sequence because partial sequences or local spatial structures mainly mediate their biological functions. The speed of base change in lncRNA sequences exceeds the evolutionary time scale. It follows that lncRNAs evolve faster than protein coding genes, suggesting that nucleotide sequence conservation is not essential for preserving lncRNAs functionality (150, 151). lncRNAs follow different conservation criteria than those of protein coding genes (150, 152). Identity concentrates on short sections and the secondary structure, unlike coding genes that focus on conservation in all their length to preserve the open reading frame and ensure similarity in amino acid sequence (150). In order to find these conserved segments, diverse groups have generated tools that allow us to study their evolution and to estimate the functional conservation of lncRNAs across species, for example PLAR (153), Gencode V7 (154), Lncipedia (12), and ZFLNC (11). Currently, there are databases focused mainly on zebrafish lncRNAs annotation and expression profiles, such as ZFLNC and zflncRNApedia, for a total of 13,604 genes that transcribe 21,128 lncRNAs (11), of which 1,890 are conserved in human.

One of the most important characteristics observed in the expression patterns of zebrafish lncRNAs is the strong dynamics of temporal expression compared to protein-coding genes (155). This feature is undoubtedly relevant to consider these molecules as indicators or markers in disease processes. Also, it has been found that not only specific gene regions of the lncRNAs are conserved, but there is also significant conservation into the upstream regulatory regions (156), and the epigenetic regulatory mechanisms in the lncRNAs between zebrafish and human (155), suggesting that additional conserved non-coding RNAs have not been identified. Knockdown assays have revealed functional conservation between zebrafish and human lncRNAs. For example, morphological defects generated by *Cyrano* and *Megamind* knockdown in zebrafish (lncRNAs involved in the development of the nervous system) were rescued with mature RNA from their corresponding human orthologous (157). Similarly, *Tuna* knockdown resulted in fish with motor and locomotion defects revealing functional conservation with their human counterpart, known as lncRNAs involved in Huntington’s disease (158). These results suggest that despite the evolutionary distance between zebrafish and humans, and the discrete conservation of these molecules in sequence, lncRNAs are essential in homeostasis and health maintenance throughout evolution.

Given the functional and expression conservation of zebrafish and human lncRNAs, these molecules are likely to play a crucial role in developing cancer in zebrafish. We found 15 lncRNAs annotated in the zebrafish genome, orthologous to 18 human lncRNAs associated with breast cancer, using the Gencode (154), Lnc2Cancer v2.0 (30), LNCipedia (12), and ZFLNC (11) databases. The 18 human lncRNAs conserved in the zebrafish genome participate in oncogenic processes such as cell cycle control, proliferation, differentiation, migration, invasion, metastasis, angiogenesis and maintenance of cancer stem cells, according to CancerSEA (http://biocc.hrbmu.edu.cn/CancerSEA), LncTarD and Lnc2Cancer v2.0 data (**Table 2**).

**Table 2:** Functional relationship between conserved lncRNAs and breast cancer hallmarks.

Zebrafish name	Human lncRNA name	lncRNA type	Activate	Inhibited	O or S	Expression
ZFLNCT16634 (LOC103909273 un-characterized)	DLX6-AS1	Antisense	P, I, EMT, Mi, T	Ap, CellC, D, DNAd, DNAr, H, Inf, Q	O	Up-regulated
ZFLNCT02505 (ENSDART00000153684.2, CR751227.1-201)	HAGLR (HOXD-AS1)	Antisense,	An, D, Inf, Me	DNA r, I, Q	O	_
ZFLNCT08532 (NONDRET012204.1)	HOTAIR	Antisense, lincRNA	D, Mi, Me, I, T	DNAr, EMT, I, Ap	O	Up-regulated
ZFLNCT02498 (ENSDART00000155072.3, ENSDART00000155419.2, ENSDART00000155896.2, LOC103910246)	HOXA11-AS	Antisense, sponge	CellC, Inf, Q, P, I, Me, Mi	DNAd, DNAr, I, Ap	O/S in OC	Up-regulated
	HOXA-AS2	Antisense, ceRNA, sponge	Ch, P, I, Mi, T, Prog, EMT	D, Ap	O	–
	HOXA-AS3	Antisense, sponge	P, Mi, I, S, Me	EMT, Inf, Me, An, Ap	O	Up-regulated
	HOXB-AS1	Antisense, ceRNA	P, CellC, I, Mi	Ap	O	Up-regulated
ZFLNCT19656 (si:dkey-81p22.11)	HOXC-AS3	Sponge	P, H, T, I, Mi, Me	Q	O	Up-regulated
ZFLNCT01281	LINC00649	Antisense		An, Ap, cellC, D, EMT, H, I, Me, P, Q	_	Down-regulated
ZFLNCT17432 (ENSDART00000153409.2)	LINC00324	lincRNA	P, I, Me, Mi, S	Ap	O	Up-regulated
ZFLNCT14004 (ENSDART00000149569.3)	LINC00461	lincRNA, ceRNA, Sponge	P, cellC, DNAd, S, Mi, I, Me, T, EMT	An, H, Q, Ap	O	Up-regulated
ZFLNCT12716 (lnc2_zgc:194285, lnc1_zgc:194285)	MALAT1	Sponge, ceRNA, lincRNA	Mi, I, EMT, An, H, S, P, Me, Ap	An, CellC, DNAd, DNAr, EMT, Inf, I, Me, Q, P	O and S	_
ZFLNCT01941 (lnc_ghrhra)	MAPT-AS1	Antisense	CellC, S. In ER negative BRCA induce P and Mi.	Ap, DNAd, DNAr, EMT, H, I, Me, P, Q	S	_
ZFLNCT11804 (oip5-as1-202, Cyrano)	OIP5-AS1 (Linc-OIP5, Cyrano)	ceRNA	P, Mi, I, T, CellC, DNAd, S, Me, EMT	P, RR (CRC)	O ans S	Up-regulated
ZFLNCT11748 (NONDRET002400.1)	PAX8-AS1	Processed transcript	Ap	Ap, CellC, DNAd, DNAr, H, Inf, I, Me, P, Q, S	S	Down-regulated
ZFLNCT19020 (PF102167.1-201, ENSDART00000149948.3, sox2ot, si:ch73-334e23.1, LOC101883930)	SOX2-OT	Sense overlapping	P, I, Me	Ap, CellC, D, DNAd, DNAr, H, Q	O	Up-regulated
ZFLNCT09180 (BX571737.1-201)	TTN-AS1	Antisense, ceRNA, sponge, lincRNA	DNAd, P and Me in ESCC. P, I, EMT and Mi in BRCA and CRC	Ap, CellC, DNAd, DNAr, EMT, H, Inf, I, Me	O	Up-regulated
PTENP1	PTENP1	ceRNA, sponge	Ap	Mi, P, I	S	Down-regulated

P, proliferation; An, angiogenesis; Me, metastasis; CellC, Cell cycle; D, differentiation; DNAd, DNA damage; H, Hypoxia; Inf, Inflamation; Q, quiescence; I, Invasion; Mi, Migration; EMT, Epithelial-Mesenchymal transition; T, Tumorigenesis; S, Stemness; Ch, Chemoresitance; Prog, Progression; DR, Drug resistance; RR, Radio resistance; BRCA, Breast cancer; O, oncogenic roll; S, Tumor suppressor roll.

Zebrafish offer experimental advantages for manipulating of gene expression, facilitating the study of functional aspects of genes. Altering the expression of conserved lncRNAs in zebrafish by knockdown with morpholinos or genomic editing by CRISPR cas9, will allow the study of the non-autonomous functions of lncRNAs in the tumor microenvironment during breast cancer xenotransplantation trials, as previously carried out in the melanoma cell xenotransplants (131).

## Conclusion

Addressing breast cancer in a comprehensive manner, which involves early diagnosis through sensitive tumor markers and advancing personalized treatment design, are two of the most critical challenges in breast cancer medicine and biomedical research. Breast cancer study through xenotransplantation in zebrafish is a valuable tool given the speed with which tumors are obtained and experiments are concluded. Transplantation of human breast cancer cells in fish allows the discriminated and efficient study of aspects related to disease development, such as tumorigenesis, migration, metastasis, angiogenesis and response to drugs.

Today, these assays are mainly used to validate *in vitro* assays; however, the significant finding of conservation of zebrafish’s lncRNAs and their expression change during the zebrafish cancer process broadens the perspective. We propose that this *in vivo* model will allow the study of the functional impact of lncRNA dysregulation in the host microenvironment allowing a simultaneous search for new breast cancer-associated lncRNAs through transcriptional studies. The fine dynamics of lncRNAs expression and their relationship with the alteration of the tumor microenvironment show that these molecules are excellent candidates for the prediction and prognosis assays and possible therapeutic targets in the area of drug development.

The advantages of xenotransplantation experiments in zebrafish compared to other models suggest their potential in the personalized approach to the breast cancer treatment. One of the main strategies is zPDXs modeling, and subsequent drug screening. Subtle variations in lncRNAs expression could effectively predict the response of cancer cells to drugs and may in turn serve as new targets in the development of new treatments. The combination of the ease of performing drug screening, including lncRNAs targeted drugs, on zebrafish embryos after xenotransplantation, and the possibility of evaluating the functional *in vivo* response through the real time microscopy study, increases the robustness of xenotransplantation models. In addition, the conservation in cancer-related pathways between humans and zebrafish and the existence of interspecies molecular crosstalk during xenotransplantation support the use of knockdown and knockout zebrafish for conserved lncRNAs to determine the nature of the molecular pathways that respond to lncRNAs signaling. In the same way, xenotransplantation assays on a knockout or overexpressing cancer-related lncRNAs in zebrafish could reveal the non-autonomous function of lncRNAs in the tumor microenvironment. Besides, drug-screening trials targeting zebrafish lncRNAs or related pathways are another area that could benefit from xenotransplantation assays. An additional benefit is that the zebrafish transparency will elucidate the relationship of each of the cancer cellular phenotypes (migratory, proliferative, angiogenic) with specific zebrafish lncRNAs expression, facilitating the interpretation and analysis of the results.

## Author Contributions

CZ reviewed the literature, wrote the initial abstract, drafted the manuscript, figures, and tables, and revised the second draft following feedback from JM-Z. WAM-F reviewed the lncRNAs databases, and contributed to the second draft review. JM-Z reviewed the literature, proposed an article outline, contributed sections of the initial draft, and made editorial suggestions for the second draft. All authors contributed to the article and approved the submitted version.

## Funding

CZ was supported by a post-doctoral fellowship from the CONACYT grant A1-S-8462, and this study is part of her postdoctoral work. The work at JM-Z laboratory is supported by CONACYT grant A1-S-8462.

## Conflict of Interest

The authors declare that the research was conducted in the absence of any commercial or financial relationships that could be construed as a potential conflict of interest.

## Supplementary Material

Supplementary Table 1 Growing scientific output of work on breast cancer modeling by xenotransplantation in zebrafish.
